# Comment on ‘Impact of Resistance Training and Chicken Intake on Vascular and Muscle Health in Elderly Women’ by Fujie et al.

**DOI:** 10.1002/jcsm.13768

**Published:** 2025-03-04

**Authors:** Jiawei Du, Jinghua Hou

**Affiliations:** ^1^ Key Laboratory of Sports and Physical Fitness of the Ministry of Education Beijing Sport University Beijing China; ^2^ Department of Exercise Physiology Beijing Sport University Beijing China; ^3^ Department of Exercise Biochemistry Beijing Sport University Beijing China

Dear Editor,

We carefully read the article by Fujie et al., titled ‘Impact of resistance training and chicken intake on vascular and muscle health in elderly women’ [1], published in the *Journal of Cachexia, Sarcopenia and Muscle*. The study investigated the effects of moderate‐to‐high‐intensity resistance training combined with a high‐protein diet (steamed chicken breast) on muscle mass, strength and arterial stiffness in elderly women. The findings suggest that high‐protein intake significantly mitigates the resistance training‐induced increase in arterial stiffness while further enhancing muscle health indicators, offering critical insights into clinical and public health implications. However, upon in‐depth analysis, we believe that several key limitations in the study design, methodology, mechanistic exploration and statistical analysis may affect the reliability of the conclusions and their clinical translational value.

First, the study employed carotid‐femoral pulse wave velocity (cfPWV) and carotid β‐stiffness as the primary indicators of arterial stiffness, whereas muscle mass was assessed via ultrasound measurements of muscle thickness and echo intensity (EI). However, the descriptions of these measurement methods are entirely absent. For instance, the specific equipment used for cfPWV, the exact locations for signal acquisition and the standardized procedures for calculating time differences were not provided. Similarly, the ultrasound measurements of muscle thickness and EI lacked details on probe frequency, participant positioning and standardized protocols for repeated measurements. This lack of methodological detail may hinder the reproducibility of the study by other researchers and undermine the credibility and generalizability of the findings. We recommend supplementing the study with detailed descriptions of experimental procedures and referencing internationally recognized standards (e.g., guidelines from the European Society of Hypertension or the American Institute of Ultrasound in Medicine) to ensure transparency and reproducibility.

Second, the study did not assess the potential impact of high‐protein intake on renal function (e.g., glomerular filtration rate [GFR]) or discuss the potential risks of long‐term high‐protein consumption, such as hyperuricemia or metabolic acidosis. Whereas high‐protein diets have significant benefits for muscle health, long‐term high‐protein intake may impose additional renal burden, particularly in elderly populations with potentially declining renal function [2]. The nitrogenous waste produced by protein metabolism requires renal excretion, and prolonged high‐protein intake may accelerate renal function decline. Studies have shown that high‐protein diets may increase the risk of renal function deterioration in patients with chronic kidney disease (CKD) [3]. Additionally, high‐protein diets may lead to metabolic acidosis, especially in the elderly, as the acidic byproducts of protein metabolism may exceed the body's buffering capacity, resulting in decreased blood pH [4]. Future studies should evaluate the effects of high‐protein intake on renal and metabolic health and explore individualized nutritional strategies to maximize benefits while minimizing risks.

Third, the study only mentioned angiotensin II (Ang II) as a potential mechanism for the resistance training‐induced increase in arterial stiffness but did not explore other underlying mechanisms, such as oxidative stress and inflammation. Previous research has shown that oxidative stress and inflammation are significant drivers of arterial stiffness [5]. Resistance training may increase reactive oxygen species (ROS) production, leading to oxidative stress and endothelial cell damage, thereby exacerbating arterial stiffness [6]. Ang II, through the activation of NADPH oxidase, increases ROS production, contributing to oxidative stress and vascular damage [5]. However, the study did not measure markers of oxidative stress and inflammation (e.g., MDA, SOD, CRP and IL‐6), which could provide valuable insights into the pathways by which resistance training impacts vascular health. These markers could have been measured using the existing serum samples. Furthermore, elevated Ang II levels may impair endothelium‐dependent vasodilation by reducing nitric oxide (NO) bioavailability, further exacerbating vascular stiffness. By incorporating measurements of oxidative stress and inflammatory markers and exploring the interactions between Ang II and other mechanisms, the study could provide a more comprehensive understanding of the effects of resistance training and high‐protein intake on vascular health.

In terms of statistical analysis, the study performed multiple group comparisons but did not clearly state whether comprehensive corrections for multiple comparisons were applied. Additionally, the study reported only *p* values without effect sizes (e.g., Cohen's *d* or η^2^) or 95% confidence intervals, which may lead to an overreliance on statistical significance while neglecting the clinical significance of the intervention effects. Reporting effect sizes would help clarify the magnitude of the intervention effects and better assess their translational value. Although the Bonferroni correction is conservative, it may increase the risk of Type II errors (false negatives). More flexible multiple comparison correction methods, such as Tukey HSD or Holm correction, may be more suitable for this type of study. We recommend enhancing the rigour of statistical analysis and the interpretability of the results.

Despite these limitations, Fujie et al.'s study is innovative in exploring the synergistic effects of resistance training and high‐protein diets on muscle and vascular health in elderly populations. The findings provide preliminary evidence for comprehensive intervention strategy to improve muscle health and mitigate adverse vascular effects induced by resistance training. We look forward to the authors' responses to these concerns and hope that future research will comprehensively explore the long‐term effects of resistance training and high‐protein diets in elderly populations, thereby enhancing the scientific validity and translational value of such studies.

## Author Contributions

Conceptualization: Jiawei Du and Jinghua Hou. Writing – original draft: Jiawei Du. Writing – review and editing: Jinghua Hou. All authors have read and consented to the publication of this manuscript.

## Conflicts of Interest

The authors declare no conflicts of interest.
